# Fine particulate matter 2.5 induces susceptibility to *Pseudomonas aeruginosa* infection via expansion of PD-L1^high^ neutrophils in mice

**DOI:** 10.1186/s12931-023-02640-x

**Published:** 2024-02-14

**Authors:** Li Luo, Manling Jiang, Ying Xiong, Anying Xiong, Lei Zhang, Dehong Wu, Yao Liu, Qin Ran, Jiliu Liu, Yi Zhang, Jiahuan Li, Xiang He, Junyi Wang, Guoping Li

**Affiliations:** 1 Laboratory of Allergy and Precision Medicine, Department of Pulmonary and Critical Care Medicine, Chengdu Institute of Respiratory Health, The Third People’s Hospital of Chengdu, Affiliated Hospital of Southwest Jiaotong University, Chengdu, 610031 China; 2 State Key Laboratory of Quality Research in Chinese Medicine, Macau University of Science & Technology, Macao Special Administrative Region, Taipa, China; 3 Department of Pulmonary and Critical Care Medicine, Sichuan Friendship Hospital, Chengdu, China; 4 North Sichuan Medical College, Nanchong, China

**Keywords:** PM2.5, PD-L1^high^ neutrophils, Phagocytic function, *Pseudomonas aeruginosa*, Infection

## Abstract

**Background:**

Exposure to PM2.5 has been implicated in a range of detrimental health effects, particularly affecting the respiratory system. However, the precise underlying mechanisms remain elusive.

**Methods:**

To address this objective, we collected ambient PM2.5 and administered intranasal challenges to mice, followed by single-cell RNA sequencing (scRNA-seq) to unravel the heterogeneity of neutrophils and unveil their gene expression profiles. Flow cytometry and immunofluorescence staining were subsequently conducted to validate the obtained results. Furthermore, we assessed the phagocytic potential of neutrophils upon PM2.5 exposure using gene analysis of phagocytosis signatures and bacterial uptake assays. Additionally, we utilized a mouse pneumonia model to evaluate the susceptibility of PM2.5-exposed mice to *Pseudomonas aeruginosa* infection.

**Results:**

Our study revealed a significant increase in neutrophil recruitment within the lungs of PM2.5-exposed mice, with subclustering of neutrophils uncovering subsets with distinct gene expression profiles. Notably, exposure to PM2.5 was associated with an expansion of PD-L1^high^ neutrophils, which exhibited impaired phagocytic function dependent upon PD-L1 expression. Furthermore, PM2.5 exposure was found to increase the susceptibility of mice to *Pseudomonas aeruginosa*, due in part to increased PD-L1 expression on neutrophils. Importantly, monoclonal antibody targeting of PD-L1 significantly reduced bacterial burden, dissemination, and lung inflammation in PM2.5-exposed mice upon *Pseudomonas aeruginosa* infection.

**Conclusions:**

Our study suggests that PM2.5 exposure promotes expansion of PD-L1^high^ neutrophils with impaired phagocytic function in mouse lungs, contributing to increased vulnerability to bacterial infection, and therefore targeting PD-L1 may be a therapeutic strategy for reducing the harmful effects of PM2.5 exposure on the immune system.

**Supplementary Information:**

The online version contains supplementary material available at 10.1186/s12931-023-02640-x.

## Introduction

Air pollution poses a serious global public health threat [1]. Particulate matter (PM) is among the most harmful air pollutants, including PM10, PM2.5, and ultrafine particles. PM10 particles are coarse, with an aerodynamic diameter of less than 10 μm, while PM2.5 particles are fine, with a diameter less than 2.5 μm. Ultrafine particles have a diameter less than 0.1 μm. The negative impacts of PM on human health depend on their size and chemical composition. PM10 particles tend to accumulate in the nasal cavities and trachea. PM2.5 particles are small enough to evade airway cilia and mucociliary clearance, leading to direct entry into the bronchi and penetration deep into pulmonary alveoli [2]. Exposure to a 10 µg/m^3^ increment in PM2.5 was associated with a 1.14% increase in the risk of all-cause mortality [3]. Moreover, exposure to PM2.5 is positively associated with respiratory causes of death and hospitalizations for respiratory diseases [4].

More than 90% of the world’s population is exposed to air pollution levels that exceed the World Health Organization guidelines [5]. Low-to-middle income countries have the highest levels of PM2.5 exposure and the greatest burden of respiratory infection [6]. Clinical studies have found a positive correlation between exposure to PM2.5 and the frequency of outpatient visits, emergency visits, and hospitalizations due to acute upper or lower respiratory infections [7–9]. PM2.5 has emerged as a great risk factor for respiratory infection-related death [10]. Nevertheless, the mechanisms underlying the observed increased risk of respiratory infection associated with PM2.5 exposure remain elusive.

Neutrophils, play a crucial role in the immune response to foreign particles or pathogens. Exposure to PM2.5 particles can result in the activation and infiltration of neutrophils into the affected lung tissue [11]. The excessive activation and infiltration of immune cells can contribute to further tissue damage and exacerbate the inflammatory response, emphasizing the importance of tightly regulating the function of immune cells. A key regulator in this process is the programmed death ligand 1 (PD-L1) and programmed cell death protein 1 (PD-1) immune checkpoint pathway, which plays a crucial role in preventing excessive tissue destruction during inflammation [12]. However, the upregulation of PD-L1 on immune cells can lead to local immune suppression and impaired host defense. PD-L1 molecules on neutrophils can interact with their receptors on T cells, triggering inhibitory signaling pathways that dampen T cell responses and compromise effective anti-tumor defense [13]. A neutrophil subset with high levels of PD-L1 expression has been identified in mice by the advanced single-cell RNA sequencing (scRNA-seq) technology, exerting immunosuppressive effect in sepsis [14]. In this study, we exposed mice to PM2.5 and observed an expansion of PD-L1^high^ neutrophil subset with impaired phagocytic function, leaving the host susceptible to bacterial infection such as *Pseudomonas aeruginosa* (*P. aeruginosa*). The current study suggests that focusing on PD-L1 as a target holds promise as a therapeutic strategy to decrease vulnerability to bacterial infections in individuals who have been exposed to PM2.5.

## Results

### PM2.5 exposure promotes neutrophil infiltration in mouse lung

**Fig. 1:**
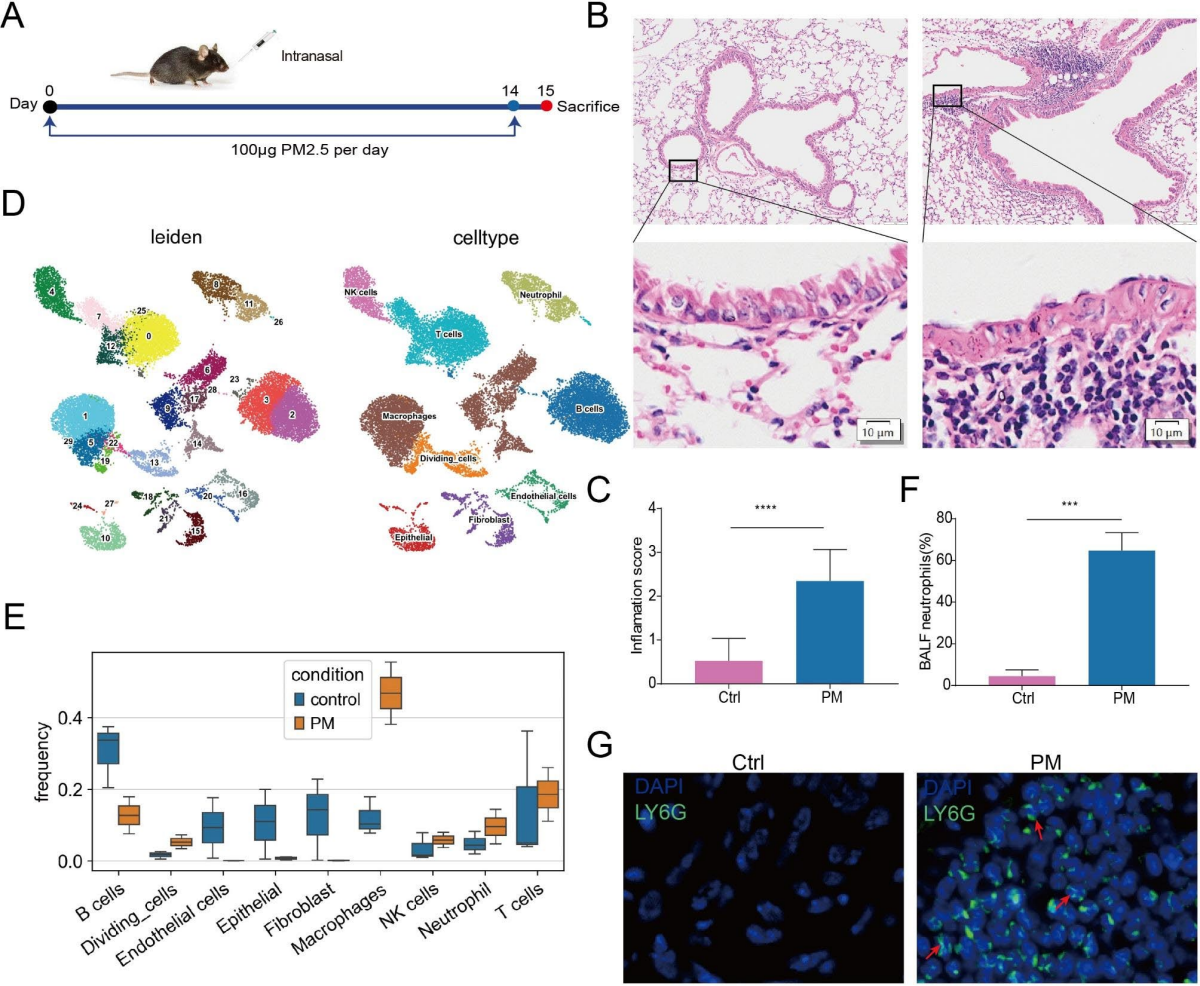
PM2.5 exposure promotes neutrophil infiltration in mouse lung. (**A**) Schematic of the experimental model. (**B**) Representative histological images of lungs by H&E staining. (**C**) Inflammation scores estimated from lung tissues with H&E staining. (**D**) UMAP visualization of 30 cell clusters (left) and 9 cell types in the lungs. (**E**) Boxplot showing the relative percentage of cell types across groups. (**F**) The relative percentage of neutrophils in the BALF. (**G**) Immunofluorescence images of LY6G in mouse lung tissues. The data are shown as means ± SEM. * *p* < 0.05, ** *p* < 0.01, *** *p* < 0.001, **** *p* < 0.0001

To investigate the effects of PM2.5 on the lungs, we collected ambient PM2.5 and intranasally challenged mice, as in our previous study (Fig. 1A). After 14 days of continuous exposure, we observed an increase in lung inflammatory infiltration compared to control mice (Fig. 1B-C). To elucidate the inflammatory cell types present in the lungs following PM2.5 exposure, we conducted scRNA-seq of lung tissues from 2 PM2.5-exposed mice and 2 control mice. After careful quality control measures, a total of 23 401 lung cells were analyzed. Nine distinct cell types were identified based on marker gene expression (Fig. 1D), with frequency analysis revealing a marked recruitment of macrophages and neutrophils in the lungs following PM2.5 exposure (Fig. 1E). Furthermore, the percentage of neutrophils in bronchoalveolar lavage fluid (BALF) significantly increased upon PM2.5 exposure (Fig. 1F), confirming our scRNA-seq findings. In the meantime, the recruitment of neutrophils in response to PM2.5 exposure was further verified using immunofluorescence staining (Fig. 1G). Taken together, these findings demonstrate that exposure to PM2.5 promotes the infiltration of neutrophils in the mouse lung.

### PM2.5 exposure promotes expansion of PD-L1^high^ neutrophils

**Fig. 2:**
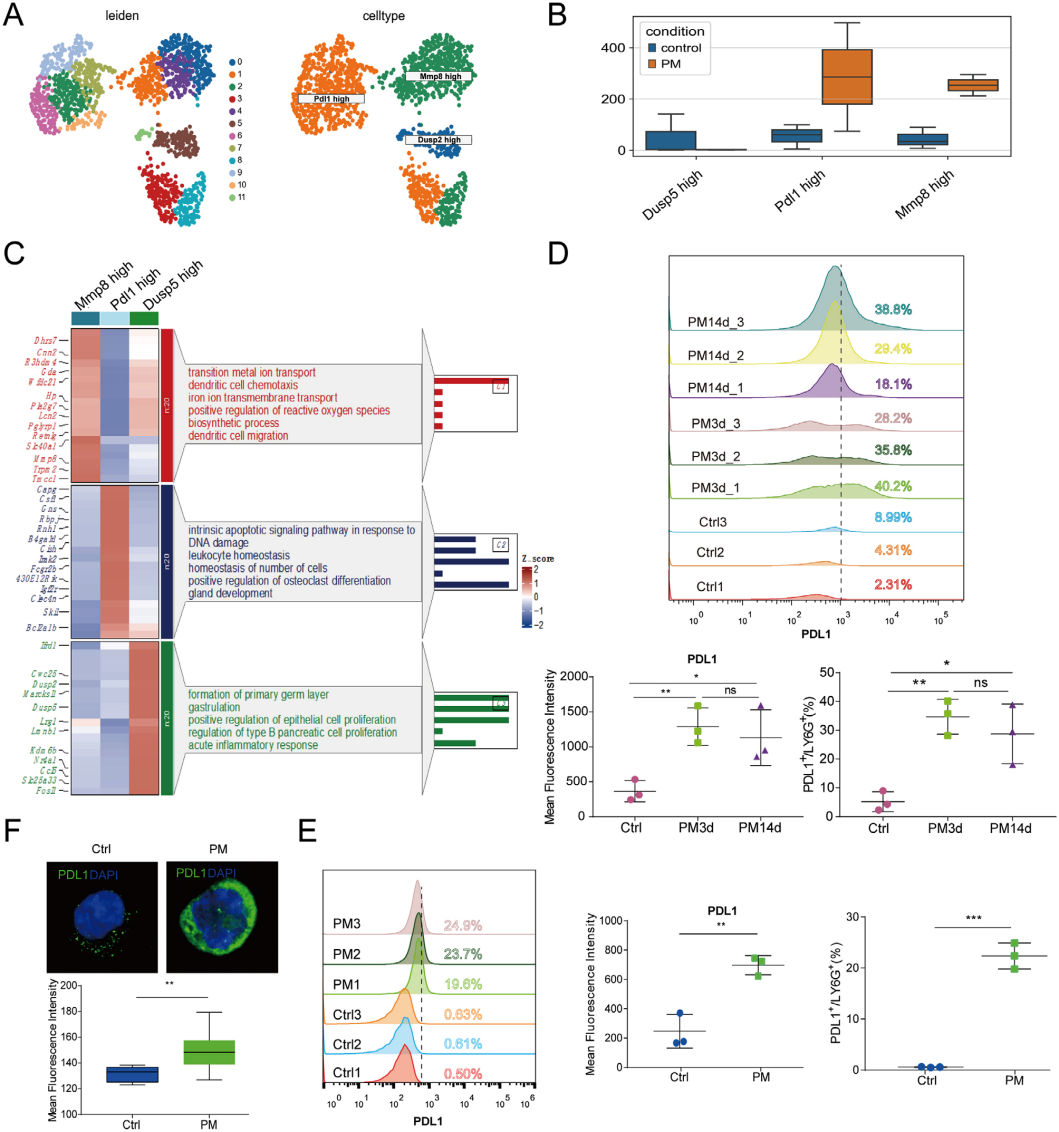
PM2.5 exposure promotes expansion of PD-L1^high^ neutrophils. (**A**) UMAP visualization of 12 neutrophil subpopulations (left) and 3 neutrophil subsets in the lungs. (**B**) Boxplot showing the counts of each neutrophil subsets across groups. (**C**) Representative differentially expressed genes and GO enrichment pathways for each subset. (**D**) Flow cytometric analysis of PD-L1^high^ neutrophils in the lungs: representative plots of PD-L1^high^ neutrophils (upper), and quantifications are depicted as MFI (lower left) and percentage (lower right). (**E**) Flow cytometric analysis of PD-L1^high^ cells in isolated neutrophils: representative plots of PD-L1^high^ neutrophils (left), and quantifications are depicted as MFI (middle) and percentage (right). (**F**) Immunofluorescence images for PD-L1 expression on isolated neutrophils (upper) and MFI for PD-L1 staining (bottom). The data are shown as means ± SEM. * *p* < 0.05, ** *p* < 0.01, *** *p* < 0.001, **** *p* < 0.0001

In order to investigate the impact of PM2.5 exposure on neutrophil heterogeneity, we conducted subclustering of neutrophils (1523 cells) from our scRNA-seq dataset and identified 12 subpopulations that could be classified into three major groups based on differentially expressed genes: *Mmp8*^high^ subset, *Pdl1*^high^ subset, and *Dusp5*^high^ subset (Fig. 2A). Interestingly, the number of cells in both the *Pdl1*^high^ and *Mmp8*^high^ subsets was significantly higher in PM2.5-exposed mice compared to control mice (Fig. 2B). Functional enrichment analysis of the *Mmp8*^high^ subset revealed significant up-regulation of metal ion transport, dendritic cell chemotaxis, and ROS pathways, while the *Pdl1*^high^ subset was predominantly enriched in apoptotic and cell homeostasis pathways (Fig. 2C).

The upregulation of PD-L1 on neutrophils have been shown to play a critical role in regulating immune responses. Thus, we assessed the expression of PD-L1 (encoded by *Pdl1*) at the protein level using flow cytometry and observed a significant increase in the number of PD-L1^high^ neutrophils in the lungs of mice following 14 days of continuous exposure to PM2.5 (Fig. 2D). Surprisingly, we found that the expansion of PD-L1^high^ neutrophils was already present on the third day of exposure (Fig. 2D). To confirm these findings, we isolated neutrophils from the bone marrow of healthy adult C57BL/6 mice and performed both flow cytometry (Fig. 2E) and immunofluorescence (Fig. 2F) analyses, which demonstrated a significant increase in PD-L1 expression on neutrophils following PM2.5 (62 µg/cm^2^) stimulation. Taken together, our results suggest that PM2.5 exposure promotes the expansion of PD-L1^high^ neutrophils in lungs.

### PM2.5 exposure impaired the phagocytic function of neutrophils in a PD-L1–dependent manner

**Fig. 3:**
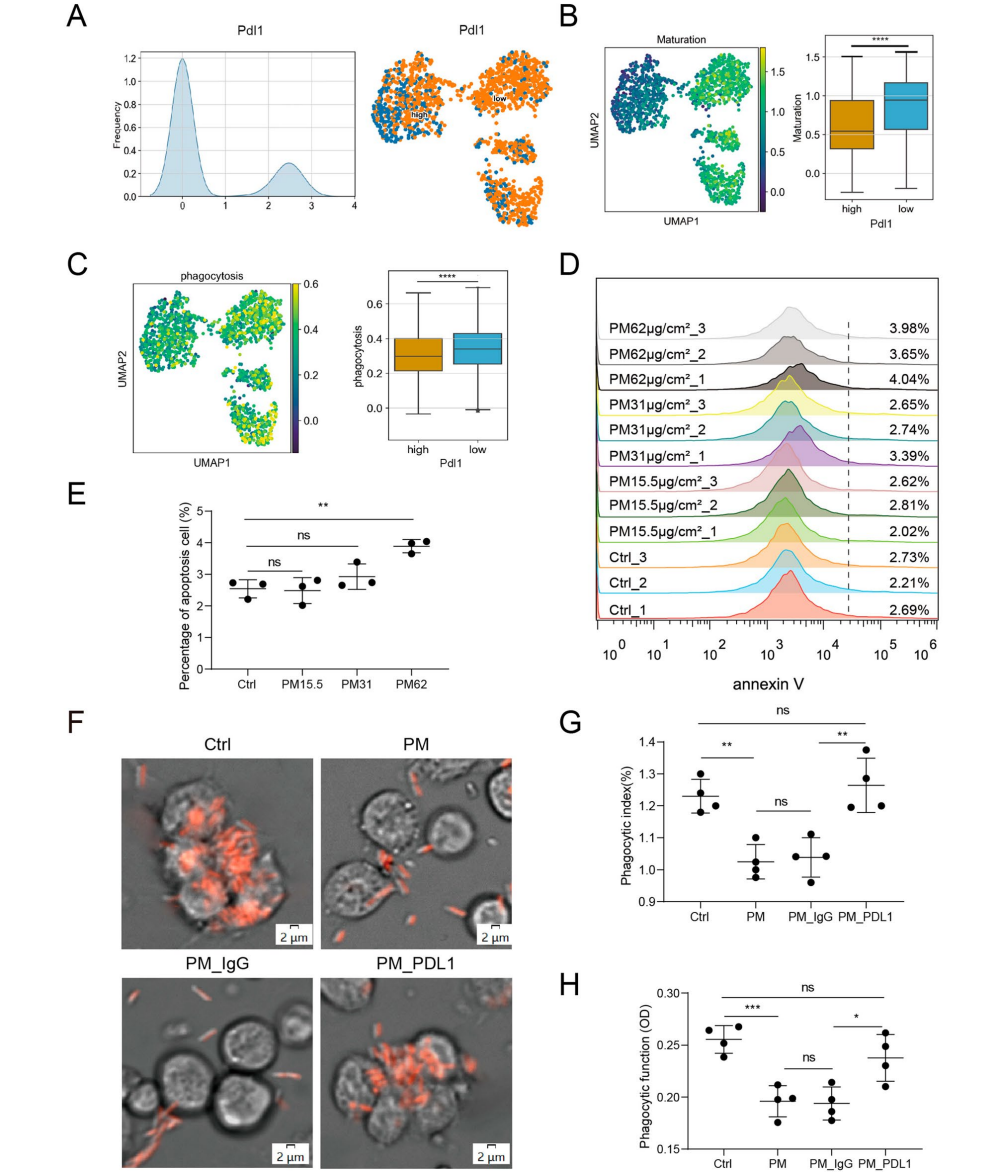
PM2.5 exposure impaired the phagocytic function of neutrophils in a PD-L1–dependent manner. (**A**) Ridge plot (left) and UMAP plot (right) showing the distribution of high and low Pdl1 expression with a threshold of 1. (**B-C**) The UMAP maps showing AUC scores of selected gene signatures (left), and box plots showing AUC scores of selected gene signatures. (**D**) Apoptosis analysis of PM2.5 treated neutrophils via flow cytometric. (**E**) Quantifications of (**D**) are depicted as percentage. (**F**) Representative images of the phagocytosis process of mCherry-PAO1 by neutrophils. (**G**) Quantifications of (**F**) are depicted as phagocytic index. (**H**) Phagocytic function of neutrophils assessed by neutral red phagocytosis assay. data are shown as means ± SEM. * *p* < 0.05, ** *p* < 0.01, *** *p* < 0.001, **** *p* < 0.0001

To shed light on the function of PD-L1 highly expressed neutrophils, we defined *Pdl1*^high^ and *Pdl1*^low^ neutrophils based on the expression levels of *Pdl1* (threshold of 1) in our scRNA-seq dataset (Fig. 3A). By calculating the phagocytosis signature gene score for each cell using AUCell, we found that *Pdl1*^high^ neutrophils exhibited significantly lower enrichment of maturation signature (Fig. 3B) and phagocytosis signature (Fig. 3C) compared to *Pdl1*^low^ neutrophils, suggesting that impaired phagocytosis is a hallmark of *Pdl1*^high^ neutrophils that were in an immature state. In order to validate our findings, we employed mCherry-tagged *P. aeruginosa* to assess the phagocytic activity of murine bone marrow neutrophils following exposure to PM2.5. Intriguingly, neutrophils exposed to PM2.5 (62 µg/cm^2^) exhibited a noteworthy reduction in bacterial uptake, as illustrated in Supplementary Fig. 1A-B. Consistent results were observed in neutral red phagocytic assays, as depicted in Supplementary Fig. 1C. However, as depicted in Fig. 2C, the PM2.5-induced Pdl1^high^ subset in lungs were found to be enriched in apoptotic pathways. Recognizing that neutrophils indeed underwent apoptosis at a concentration of 62 µg/cm2 PM2.5 (Fig. 3D-E), we opted for a lower concentration of PM2.5 (31 µg/cm^2^), which does not induce apoptosis (Fig. 3D-E) but effectively triggers the expression of PD-L1 (Supplementary Fig. 2A-C). The results still demonstrated a diminished phagocytic capacity in PM2.5-exposed neutrophils (Fig. 3F-H). Notably, the use of a PD-L1 monoclonal antibody effectively restored this phagocytic defect, presenting a compelling contrast to the isotype IgG antibody (Fig. 3F-H). Collectively, these data demonstrate that PM2.5 exposure impaired the phagocytic function of neutrophils in a PD-L1-dependent manner.

Moreover, to explore the molecular pathways implicated in PM2.5-induced inhibition of neutrophil phagocytosis by PD-L1, we performed protein–protein interaction network analysis and KEGG analysis with the differentially expressed genes (DEGs) of *Pdl1*^high^ neutrophils in our scRNA-seq dataset. Beyond the PD-L1 checkpoint pathway, our results exhibited a noteworthy enrichment of pathways such as Toll-like receptor signaling, mTOR signaling, TNF signaling, and PI3K-AKT signaling (Supplementary Fig. 3). This observation suggests a potential involvement of these pathways in the impairment of neutrophil phagocytic function mediated by PD-L1 following exposure to PM2.5.

### PM2.5-exposed mice are susceptible to *P. aeruginosa*

**Fig. 4:**
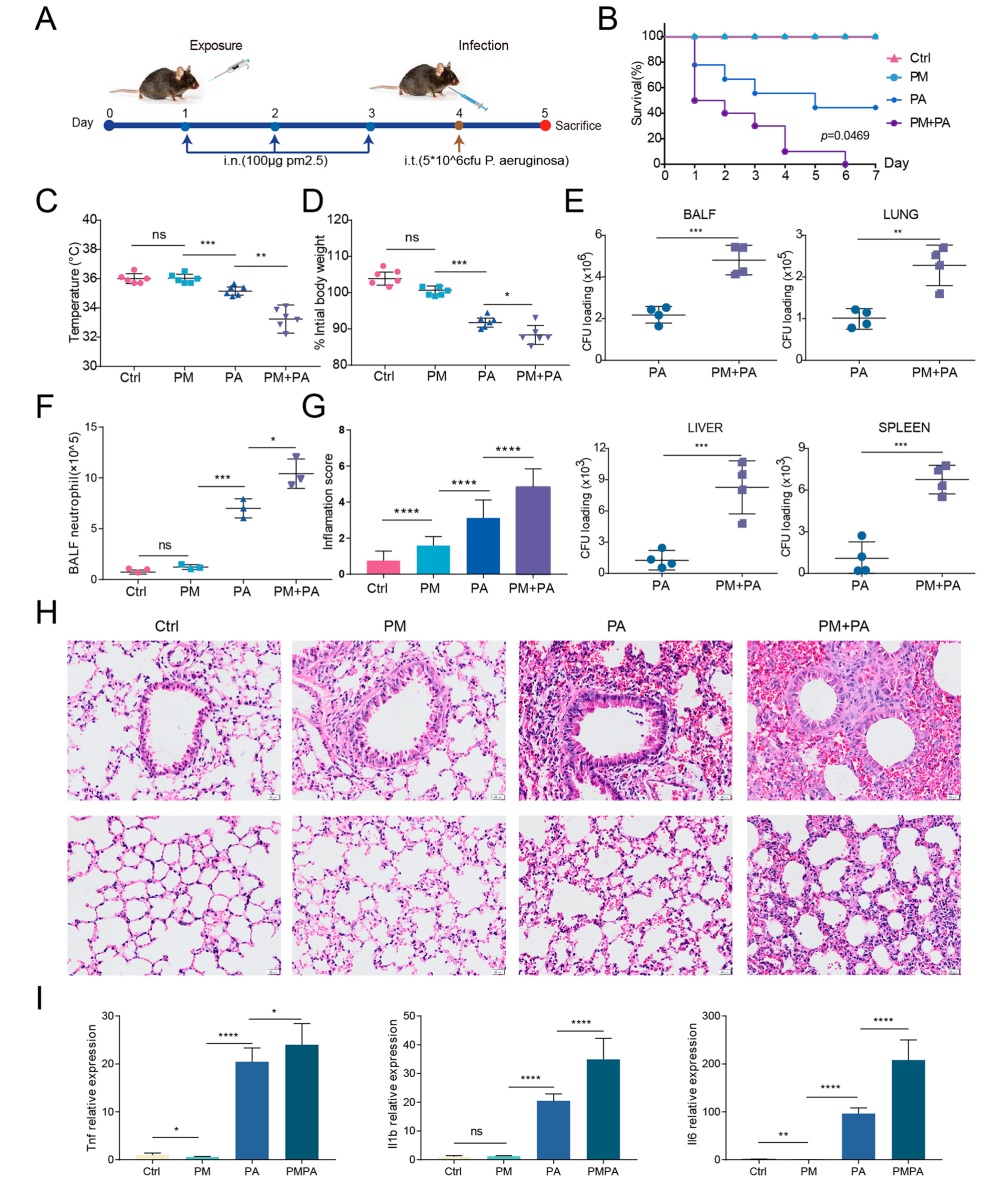
PM2.5-exposed mice are susceptible to *P. aeruginosa*. (**A**) Schematic of the experimental model. (**B**) Survival curves of mice. (**C-D**) Body temperature (**C**) and weight changes (**D**) following infection. (**E**) Bacterial burden in the BALF (upper left), lung (upper right), liver (lower left), and spleen (lower right) are shown in colony forming units (CFU). (**F**) The counts of neutrophils in the BALF. (**H**) Representative histological images of lungs by H&E staining. (**G**) Inflammation scores estimated from lung tissues with H&E staining. (**I**) Expression of *Tnf* (left), *Il1b* (middle), and *Il6* (right) in mouse lungs at mRNA level. The data are shown as means ± SEM. * *p* < 0.05, ** *p* < 0.01, *** *p* < 0.001, **** *p* < 0.0001

Neutrophils are the primary phagocytes that form the first line of cell-mediated host defense against bacterial infection, leading us to hypothesize that PM2.5 exposure may increase susceptibility to bacteria. Since we observed a significant increase in the number of PD-L1^high^ neutrophils as early as 3 days following PM2.5 exposure (Fig. 2D), we test this hypothesis by utilizing a mouse pneumonia model via intratracheally injecting PAO1 (5 × 10^6^ CFU) into mice after continuous 3 days of PM2.5 exposure (Fig. 4A). All PM2.5-exposed mice succumbed to *P. aeruginosa* infection within 6 days, while 50% of unexposed mice survived (Fig. 4B). Additionally, PM2.5 exposure resulted in increased hypothermia (Fig. 4C) and weight loss (Fig. 4D) compared to unexposed mice during infection. Furthermore, PM2.5-exposed mice displayed impaired bacterial clearance in the lungs and BALF, with increased dissemination into the liver and spleen after infection, compared to unexposed mice (Fig. 4E). We also noted an elevated infiltration of inflammatory cells and higher levels of acute inflammation in lung tissues in PM2.5-exposed mice, in addition to a marked increase in the number of neutrophils in BALF during *P. aeruginosa* infection (Fig. 4F-H). Moreover, we probed for the expression of proinflammatory cytokines mRNA, including *Tnf*, *Il1b*, and *Il6*, and found that their levels were significantly higher in the lungs of PM2.5-exposed mice upon infection (Fig. 4I). Collectively, these data suggest that PM2.5 exposure increases vulnerability to *P. aeruginosa* and aggravates inflammatory lung injury in mice upon infection.

### Blocking of PD-L1 reduces susceptibility to *P. aeruginosa* induced by PM2.5 exposure

**Fig. 5:**
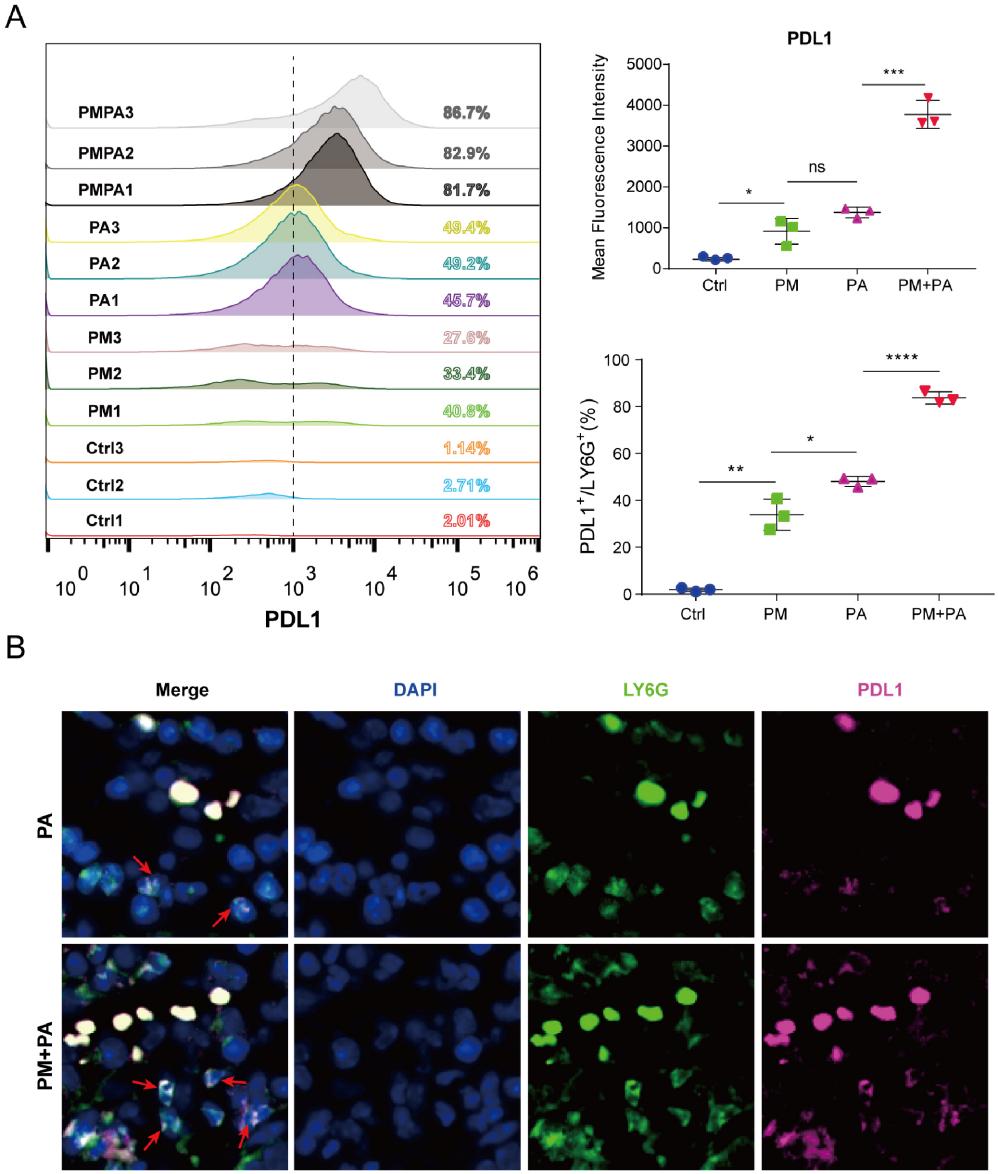
Increased expression of PD-L1 on neutrophils in PM2.5-exposed mice following infection. (**A**) Flow cytometric analysis of PD-L1^high^ neutrophils in the lungs: representative plots of PD-L1^high^ neutrophils (left), and quantifications are depicted as MFI (upper right) and percentage (lower right). (**B**) Immunofluorescence images of PD-L1^high^ neutrophils in mouse lung tissues. The data are shown as means ± SEM. * *p* < 0.05, ** *p* < 0.01, *** *p* < 0.001, **** *p* < 0.0001

Next, we employed flow cytometry to verify the expression of PD-L1 on neutrophils in our murine model of infection. Our results revealed a significantly higher proportion of neutrophils with high levels of PD-L1 in PM2.5-exposed mice compared to unexposed mice following *P. aeruginosa* infection (Fig. 5A). These findings were confirmed by lung tissue immunofluorescence, which showed an increased number of PD-L1^high^ neutrophils in PM2.5-exposed mice during infection relative to unexposed mice (Fig. 5B).

**Fig. 6:**
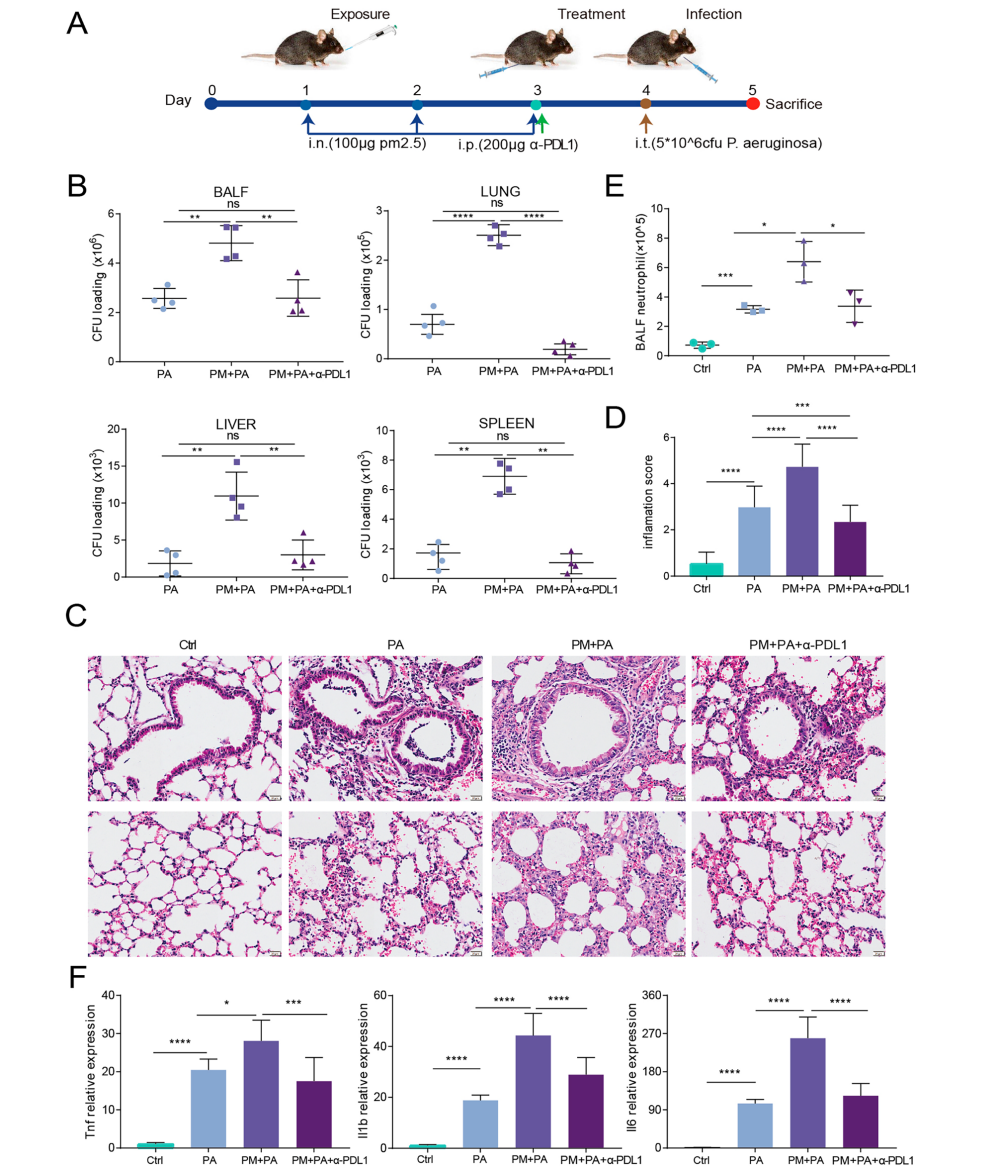
Blocking of PD-L1 reduces susceptibility to P. aeruginosa induced by PM2.5 exposure. (**A**) Schematic of the experimental model. (**B**) Bacterial burden in the BALF (upper left), lung (upper right), liver (lower left), and spleen (lower right) are shown in colony forming units (CFU). (**C**) Representative histological images of lungs by H&E staining. (**D**) Inflammation scores estimated from lung tissues with H&E staining. (**E**) The counts of neutrophils in the BALF. (**F**) Expression of *Tnf* (left), *Il1b* (middle), and *Il6* (right) in mouse lungs at mRNA level. The data are shown as means ± SEM. * *p* < 0.05, ** *p* < 0.01, *** *p* < 0.001, **** *p* < 0.0001

We then hypothesized that prophylactic treatment with anti-PD-L1 antibody would improve susceptibility to bacterial infection induced by PM2.5. Administration of anti-PD-L1 monoclonal antibody to PM2.5-exposed mice before infection (Fig. 6A) resulted in significant improvements to reduce bacterial burden and dissemination compared to PM2.5-exposed mice that received the antibody (Fig. 6B). Accordingly, anti-PD-L1 treatment was effective in reducing lung inflammation score (Fig. 6C-D), BALF neutrophil counts (Fig. 6E), and expression of proinflammatory cytokines (Fig. 6F) in PM2.5-exposed mice upon *P. aeruginosa* infection. In contrast, the isotype control antibody failed to exhibit similar improvements in bacterial burden and lung inflammatory infiltration, thereby confirming the specificity and effectiveness of the anti-PD-L1 treatment (Supplementary Fig. 4). Additionally, to investigate whether the antibody would deplete PD-L1^high^ neutrophils in mice, we conducted a flow cytometric analysis. We observed a substantial decrease in the percentage of PDL1^high^ neutrophils in mice exposed to PM2.5 and treated with a PD-L1 antibody, in contrast to mice treated with an isotype antibody, upon *P. aeruginosa* infection (Supplementary Fig. 5).

## Discussion

Neutrophils are critical in initiating an immune response to inflammation. Previous studies have shown that exposure to PM2.5 can increase neutrophil recruitment and activation in the lungs, leading to tissue damage and inflammation [11, 15]. In line with previous studies, we exposed mice to PM2.5 and observed a notable elevation in neutrophilic infiltration within lung tissues in our current study. Traditionally, neutrophils were considered as a relatively homogeneous cell population; however, recent research has uncovered significant heterogeneity within the neutrophil population in various conditions such as autoimmune diseases, sepsis, and tumor microenvironments [16–18]. Given the variations within the neutrophil population, it is reasonable to speculate that different subsets of neutrophils may have distinct roles in response to PM2.5 stimulation. Consistent with this notion, our scRNA-seq analysis showed that exposure to PM2.5 led to an increase of both *Pdl1*^high^ and *Mmp8*^high^ subsets of neutrophils, with functionally enriched pathways demonstrating unique patterns for each subset. Previous research has shown that MMP8 plays a role in the chemotaxis and migration of neutrophils during inflammation [19]. Interestingly, our findings suggest that the *Mmp8*^high^ subset was functionally enriched in metal ion transport, chemotaxis, and ROS pathways, indicating their potential contribution to the activation of inflammation and oxidative stress injury following PM2.5 exposure. However, further investigation is required to determine the specific role of MMP8^high^ neutrophils during PM2.5 exposure.

Our study showed a remarkable expansion of neutrophils expressing PD-L1 after exposure to PM2.5. We further validated this finding through flow cytometry and immunofluorescence experiments, which confirmed the upregulation of PD-L1 on neutrophils after PM2.5 stimulation. Importantly, the expression of PD-L1 by neutrophils under the influence of PM2.5 has not been previously reported. Although PD-L1 has been defined on NKT cells [20] and epithelial cells [21] upon particulate matter exposure, it has not been identified in granulocytes. The expansion of neutrophils expressing PD-L1 in response to PM2.5 exposure highlights a potential mechanism through which the immune system may respond to airborne particulate matter. The PD1/PD-L1 axis functions as a brake mechanism in restricting the immune response, preventing immune over-activation and tissue damage, and inhibiting the beneficial T-cell functions in antitumor immunity [12]. Recently, PD-L1-expressing neutrophils have gained increasing recognition due to their abundant presence and rapid responsiveness compared to other immune cells. Previous study has shown that cancer-associated fibroblasts induce PD-L1^high^ neutrophils via the IL6-STAT3 signaling pathway, promoting immune suppression in hepatocellular carcinoma [22]. Additionally, tumor-derived lactate induces the expansion of PD-L1^high^ neutrophils via MCT1/NF-κB/COX-2, inhibiting the efficacy of lenvatinib [23]. Accumulating evidence also suggests that PD-L1^high^ neutrophils play a key role not only in cancer immunomodulation, but also in inflammatory diseases. In COVID-19 cases, it has been observed that macrophage SPP1 drives the differentiation of PD-L1^high^ neutrophils, which are considered hallmarks of severe COVID-19 [24]. In sepsis, the neutrophil subset of highly expressed PD-L1 exert an immunosuppressive effect through direct contact, inhibiting T-cell activation and promoting T-cell apoptosis and trans-differentiation [14]. In the present study, our functional enrichment study showed that the *Pdl1*^high^ subset was predominantly enriched in apoptotic and cell homeostasis pathways, indicating that the upregulation of PD-L1 on neutrophils may serve as a protective mechanism during exposure to PM2.5, allowing these cells to regulate their own activity and/or modulate the activity of other immune cells in the lung microenvironment. Nevertheless, the upregulation of apoptotic pathway in *Pdl1*^high^ subset seems contradictory to the previous finding that increased PD-L1 expression on human neutrophils during sepsis delays cellular apoptosis via the PI3K-AKT pathway [25]. Further studies are needed to investigate the specific molecular pathways that regulate neutrophil apoptosis in response to PD-L1 expression during PM2.5 exposure.

Excessive accumulation of PD-L1^high^ neutrophils may also contribute to local immune suppression and impaired host defense against respiratory infections. Neutrophils constitute a crucial element of the innate immune system and represent the first line defenders in protecting the host from bacterial infections. Their phagocytic activity, which involves the internalization and elimination of invading pathogens, is one of their essential functions [26]. Recent mass cytometry study has revealed that PD-L1^high^ immature neutrophils exhibit reduced activation and phagocytic capabilities during sepsis [27]. Our study confirms this observation by demonstrating that exposure to PM2.5 impairs the phagocytic function of neutrophils, with antibody blocking indicating a dependence on PD-L1. This suggests that exposure to PM2.5 may lead to weakened protection against bacterial invasion. Intriguingly, we indeed observed that mice exposed to PM2.5 exhibited increased susceptibility to *P. aeruginosa* infection, as evidenced by decreased survival rate, pronounced hypothermia and weight loss, impaired bacterial clearance, increased dissemination into liver and spleen, as well as enhanced inflammation within lung tissues. Consistent with our findings, prior research has shown that the abundance of PD-L1-expressing neutrophils predicts prognosis in sepsis [28]. A period of vulnerability to infection after thermal injury is found to be associated with PD-L1^high^ neutrophil accumulation in the lung [29]. In animal models of sepsis, treatment with PD-L1 antibodies has been demonstrated to confer survival benefits [30], and PD-L1-deficient animals exhibit improved survival rates compared to wild-type counterparts [25]. Blockade of PD-L1 improves infection outcomes and enhances host defense against *Rhizopus arrhizus* [31]. Consistently, our study demonstrated a significant improvement in bacterial clearance and reduced inflammation following anti-PD-L1 monoclonal antibody therapy for PM2.5-exposed mice, suggesting that targeting PD-L1 may be a promising therapeutic strategy to alleviate bacterial infection vulnerability provoked by PM2.5 exposure. Further studies are necessary to fully comprehend the underlying molecular mechanisms and clinical implications of our findings.

In our study, the interplay between PD-L1 and key signaling pathways, such as mTOR and PI3K-AKT, in neutrophils following PM2.5 exposure has emerged as significant. In T cells, PD-1 has been implicated in PTEN activation, leading to the inhibition of TCR-mediated PI3K/AKT activation [32]. Conversely, the PI3K/AKT/mTOR pathway exerts control over PD-L1 expression. Dysregulated activation of the PI3K/AKT/mTOR pathway results in enhanced PD-L1 post-transcription and protein translation. For instance, heightened AKT/mTOR expression stimulates PD-L1 expression in mice, whereas inhibitors related to the PI3K/AKT/mTOR pathway decrease PD-L1 expression [33, 34]. However, recent studies have demonstrated a reciprocal relationship, showing that PD-L1 overexpression can activate the PI3K/AKT/mTOR pathway. PD‑L1 has been identified as promoting the growth of head and neck squamous cell carcinoma cells through mTOR signaling [35]. In acute myeloid leukemia, PD-L1 regulates cell proliferation by activating the PI3K-AKT signaling pathway [36]. In the context of neutrophils, PD-L1 forms complexes with the p85 subunit of PI3K, activating AKT-dependent survival signaling during sepsis [25]. Additionally, PD-L1 maintains the release of neutrophil extracellular traps by modulating autophagy through the PI3K/Akt/mTOR pathway in acute respiratory distress syndrome [37]. Interestingly, a study has highlighted that the collaboration of PM2.5 with P. aeruginosa leads to the inhibition of alveolar macrophage phagocytosis, exerting its influence through the intricate mTOR pathway. This discovery also underscores a profound connection between PM2.5 and mTOR-related pathway, elucidating their role in dampening immune cell activity [38]. Nonetheless, it’s essential to note that our findings are rooted in bioinformatics analyses, necessitating careful interpretation and underscoring the imperative requirement for experimental validation. Future investigations will play a crucial role in elucidating the upstream/downstream regulatory relationships and the molecular intricacies of these interactions, providing a more profound comprehension of the regulatory mechanisms involving PM2.5-induced PD-L1 in neutrophil function.

An intriguing observation in our study is the dramatic increase in macrophages within mouse lungs following PM2.5 stimulation. It is plausible to hypothesize that PM2.5 may also impair the functionality of these macrophages. Indeed, recent studies have highlighted that PM2.5 can suppress the phagocytic activity of macrophages, thereby worsening bacterial infections [38–40]. Moreover, a recent report has demonstrated that the expression of PD-1 by tumor-associated macrophages can hinder both phagocytosis and anti-tumor immune responses [41]. However, the question of whether PM2.5 might suppress macrophage phagocytic function by inducing PD-L1 expression remains unclear and warrants investigation in future studies.

In summary, our study provides valuable insights into the pathogenic mechanisms underlying the detrimental impact of PM2.5 exposure on respiratory health. Our results demonstrate that PM2.5 exposure induces neutrophil infiltration and PD-L1^high^ neutrophil expansion in murine lungs, which hampers neutrophil phagocytic activity via a PD-L1-dependent mechanism, thereby exacerbating susceptibility to bacterial infections like *P. aeruginosa*. Targeting PD-L1 might be a promising therapeutic strategy to reduce the risk of bacterial infection in individuals exposed to PM2.5.

## Methods

### Reagents

All reagents, antibodies and their providers are listed as follows: HE Staining Kit (Beyotime, C0105S, China), *pseudomonas* selective medium (Huankai, 027054B, China), collagenase type IV (Worthington, LS004186, America), rat anti-CD45-FITC (Invitrogen, 11-0451-82, America), rat anti-Ly6G-APC (Invitrogen, 17-9668-80, America), rat anti-CD274-PE (Invitrogen, 12-5982-81, America), 7-AAD (Bioegend, 420,403, America), rat anti-Ly6G (Invitrogen, 14-5931-82, America), mouse anti-PD-L1(Invitrogen, 14-5983-82, America), anti-fade mounting medium containing DAPI stain (Beyotime, P0131, China), Percoll cell separation solution (Beyotime, BS909, China), RBC lysis buffer (Biosharp, BL503B, China), neutral red kit (Beyotime, C0013, China), fetal bovine serum (Gibco, C0235, America), RPMI-1640 (Gibco, C11875500BT, America), IMDM (Gibco, C12440500BT, America), PD-L1 monoclonal antibody (BioXcell, BE0101, America), Isotype control Rat IgG2b κ (BioXcell, BE0090, America), Annexin V-APC/DAPI Apoptosis Kit (Elabscience, E-CK-A258B, China,).

### PM2.5-exposed murine model

All animal experiments were performed and approved in accordance with the guidelines of the Institutional Animal Care and Use Committee in Southwest Jiaotong University. Female C57BL/6 mice (18-20 g, 6–8 weeks old) were obtained from Tengxin Biotechnology Company. Upon arrival, the mice were acclimated for 1 week under specific pathogen-free (SPF) conditions. They were provided with ad libitum access to food and water and maintained under a 12-hour light-dark cycle to ensure optimal health and well-being. PM2.5-exposed model was prepared as our previously described [2]. Briefly, mice were exposed to PM2.5 (100 µg) via intranasal inhalation for continuous 14 days. On the 15th day, all mice were euthanized, and their lung tissues were collected. Bronchoalveolar lavage fluid (BAL F) was obtained using pre-cooling PBS, and the total number of cells was counted. The cells in the BALF were then classified under a microscope after Wright-Giemsa dye staining.

### Histology

The right lower lung tissue was carefully extracted from each mouse and subsequently fixed by immersing it in a 4% paraformaldehyde solution. To prepare the tissue for analysis, routine paraffin embedding was performed, and the resulting lung tissue blocks were sliced into 5-µm-thick sections using a precision microtome. Hematoxylin-eosin (HE) staining was conducted on the tissue sections, following the standardized instructions provided with the staining kit. In line with our previous method [42], we determined the inflammation score based on H&E slides. This involved scoring the severity of inflammatory cell infiltrates around airways and vessels for greatest severity (0: normal; 1: <3 cell diameter thick; 2: 4–10 cells thick; 3: >10 cells thick) and overall extent (0: normal; 1: <25% of the sample; 2: 25–75%; 3: >75%) in a blinded fashion. The index was calculated by multiplying severity by extent, with a maximum possible score of 9.

For tissue immunofluorescence analysis, the paraffin sections were initially deparaffinized and subjected to antigen retrieval. Subsequently, to prevent non-specific binding, the sections were blocked with 10% goat serum at 37 °C for 45 min. The specific marker Ly6g antibody was then employed to target and label neutrophils, and the sections were incubated overnight at 4 °C. Following this primary antibody incubation, a fluorescently labeled secondary antibody was applied and incubated at 37 °C for 1 h. To further investigate the expression patterns of interest, the PD-L1 antibody was subsequently introduced and allowed to incubate for 2 h at room temperature. Nuclei were finally stained with DAPI to enable precise cellular localization. The samples were scanned using the VS200 whole slide scanner from Olympus. The obtained digital images were then subjected to quantitative analysis utilizing the CellSens Dimension Software.

### Single-cell RNA sequencing

Lung tissue scRNA-seq was performed as our previously described [43]. Briefly, upon collection, freshly cells underwent immediate quantification and were processed using the Chromium Next GEM Single Cell 3’ Kit v2 (10x Genomics) as per the manufacturer’s instructions. Subsequently, libraries were constructed following the recommended protocols provided by Illumina. To ensure library quality, insert size was measured using a bioanalyzer from Agilent, while DNA concentration was determined using a Qubit fluorometer. The effective concentration of each library was further assessed through quantitative polymerase chain reaction. Finally, these libraries were sequenced using the NextSeq 2000 platform (Illumina). To align the sequence data to the GRCm38/91 (mm10) reference genome, obtained from the 10x Genomics official website, Cell Ranger (V6.1.1, Linux) was utilized. The resulting filtered cells and their respective counts were then subjected to analysis utilizing Scanpy (http://github.com/theislab/Scanpy). This comprehensive analysis pipeline included basic filtering (min_genes = 200), total count normalization, identification of highly variable genes, computation of the neighborhood graph, identification of marker genes, and visualizations. Gene signature sets in AUCell have been included in Supplementary Table 1. Interaction network visualization was performed using the Cytoscape.

### Flow cytometry

Cells were obtained from mouse lung tissue using a previously established enzymatic digestion method. Briefly, the lung tissue was carefully placed in RPMI on ice and subsequently cut into small fragments measuring less than 1 mm. These fragments were then subjected to a 10-minute digestion with collagenase IV in a 37℃ shaker. Following digestion, the liquid mixture was passed through a 70 μm cell strainer and centrifuged at 500 g for 5 min at 4 °C to separate the cell pellet. To eliminate red blood cells, the cell pellet was resuspended in 1 ml of RBCs lysate for 1 min at room temperature. After centrifugation, the cells were resuspended in RPMI-2%FBS, and their viability was assessed using trypan blue staining. Anti-CD45-FITC was added to label white blood cells, while anti-Ly6g-APC was used to specifically mark neutrophils. Additionally, anti-PD-L1-PE was included and incubated in the dark for 30 min. Following incubation, the cells were centrifuged and washed twice with PBS. The resulting cell pellet was resuspended in 100 µl of PBS-2%FBS, and 7AAD was added, after which the mixture was incubated for 5 min. The neutrophils were subsequently assessed using flow cytometry on the SH800S platform (Sony). The gating strategies utilized in this study are presented in the supplementary Fig. 6.

### Bacteria preparation

The PAO1 and PAO1-mCherry strains were generously provided by Xikun Zhou from the West China School of Medicine, Sichuan University. These bacterial strains were activated by shaking them overnight at 37 °C and 220 rpm. Subsequently, 1 ml of the bacterial liquid was resuspended in 5 ml of fresh LB medium and cultured with continuous shaking for approximately 2 h, ensuring that the bacteria reached the logarithmic growth phase [44]. To determine the concentration of the bacterial solution, a microplate reader was utilized to measure the optical density (OD) value at 600 nm. The conversion formula between OD value and CFU (colony forming unit) follows the established ratio of 1OD = 1 × 10^9^ CFU.

### Bone marrow neutrophil isolation

Neutrophils were obtained from the bone marrow of female C57BL/6 mice (6–8 weeks old). Neutrophils were isolated from the collected bone marrow using neutrophil separation solution with gradient centrifugation, followed by red blood cell lysis. The neutrophils were carefully washed twice with PBS. Afterwards, they were centrifuged and resuspended in IMDM-20% FBS medium. The cells were then cultured in a 37 °C incubator with 5% CO2. Flow cytometry was used to test the purity of neutrophils.

### Bacterial phagocytosis assay

Live-cell imaging was employed to observe the phagocytosis process of bacteria by neutrophils. Isolated neutrophils were evenly distributed on a glass-bottom 96-well plate, with a cell count of 10^5^ per well. Neutrophils were treated with 62 µg/cm^2^ of PM2.5 and 50 µg/ml of PD-L1 mAb for a duration of 3 h. After the PM2.5 treatment, the medium was carefully aspirated, and the cells were thoroughly washed with PBS. Antibiotic-free medium was then added to support the ongoing cellular processes. Following this, mCherry-PAO1 was introduced to the incubation process at a multiplicity of infection (MOI) of 10:1. To capture the dynamic nature of the phagocytic process, the 96-well plate was placed on an Olympus IX-83 live-cell imaging system, maintaining a constant temperature of 37 °C and a CO2 concentration of 5%. Time-lapse images were recorded at intervals of 5 minutes over a period of 3 h. As previously outlined [45], the phagocytosis index was determined as the average number of phagocytized bacteria within each neutrophil. Specifically, the phagocytosis index is calculated as follows: phagocytosis index = number of bacteria phagocytized by 100 neutrophils/100.

### Neutral red phagocytosis assay

Isolated neutrophils were seeded in 96-well plates at a density of 10^5^ cells per well. The cells were treated with 62 µg/cm^2^ of PM2.5 [46] and 50 µg/ml of PD-L1 mAb/isotype control for a duration of 3 h. After removing the supernatant, the cells were thoroughly washed with PBS to eliminate any residual substances. Subsequently, 200 µl of cell culture medium and 20 µl of neutral red staining solution was added to each well. The cells were then incubated with the neutral red dye for 2 hours, and carefully rinsed twice with sterile PBS. Following this, 200 µl of neutral red detection lysate was added to each well, and the samples were placed on a shaker at room temperature for 10 min to facilitate cell lysis. Finally, the absorbance of each well was measured using a microplate reader, with a reference wavelength set at 690 nm.

### PM2.5-exposed PA infection model

PM2.5 (100 µg in 50 µl PBS) were intranasally instilled into the female C57BL/6 mice (18-20 g, 6–8 weeks old) once per day for 3 consecutive days. For PD-L1 blocking experiments, a single intraperitoneal injection of PD-L1 monoclonal antibody (200 µg in 100 µl) or same dose of isotype control antibody was administrated on the third day of PM2.5 exposure. Subsequently, a single dose of PAO1 (5 × 10^6^ CFU in 50 µl PBS) was intratracheally injected to induce the lung infection model. Their body temperature and body weight were measured before administration and prior to euthanasia. The percentage change in body weight was calculated using the following formula: percentage of initial body-weight change = final weight / initial weight × 100. Throughout the experiment, the mice were diligently monitored for symptoms and were humanely euthanized when they became moribund. The survival rate was recorded and represented using a Kaplan-Meier curve (n = 8 for each group).

### Bacterial loading assay

Twenty-four hours after the inoculation of bacteria, the lungs, livers, and spleens were carefully collected, weighed, and placed in EP tubes containing 1 ml of PBS. To facilitate homogenization, small steel balls were added to the tubes, which were then placed in a grinder for grinding. The homogenization process involved 20 s of homogenization at a frequency of 65 Hz, repeated 10 times with 10-second intervals. The resulting tissue suspension was centrifuged at room temperature with a force of 10,000 g for 1 min. Subsequently, all the supernatant was carefully transferred to a new EP tube. Both the BALF and the tissue supernatant were subjected to serial dilution, and 100 µl of the diluents were evenly spread onto selective medium [42]. The plates containing the samples were then incubated overnight in a 37 °C incubator. Following incubation, the bacterial colonies were counted.

### Quantitative PCR analysis of RNA expression

The total RNA from the tissue samples was isolated using the FastPure Tissue Total RNA Isolation Kit (Vazyme, RC101–01). The concentration and purity of the extracted RNA were assessed using the ScanDrop system (Analytik Jena AG, GER). Subsequently, the RNA was converted into cDNA using a Reverse Transcription Kit (Vazyme, R223–01) following the manufacturer’s instructions. Quantitative PCR was performed using SYBR solution obtained from Vazyme (SQ101), adhering to the manufacturer’s protocols. Primers have been included in Supplementary Table 2.

### Apoptosis analysis

Bone marrow-derived neutrophils underwent exposure to varying concentrations (0, 15.5, 31, and 62 µg/cm²) of PM2.5 at 37℃ for a duration of 3 h. Each treatment consisted of 2 × 10^5 cells per well, with meticulous triplicate replication. Subsequent to two thorough washes with PBS, the cells underwent processing following the Annexin V-APC/DAPI Apoptosis Kit protocol. The samples were promptly subjected to analysis using the SH800S platform (Sony).

### Statistical analysis

In this study, Graphpad Prism 7 was performed to conduct the statistical analysis. The experimental data is presented as means ± standard error (SEM). Two group comparisons were analyzed by non-paired t-test. Multiple group comparisons were analyzed using one-way ANOVA. The *p*-value < 0.05 was considered to indicate a statistically significant difference.

### Electronic supplementary material

Below is the link to the electronic supplementary material.

**Supplementary Material 1**: Supplementary Fig. 1: Representative images of the phagocytosis process of mCherry-PAO1 by neutrophils. (A) Quantifications of (B) are depicted as phagocytic index. (C) Phagocytic function of neutrophils assessed by neutral red phagocytosis assay. data are shown as means ± SEM. * *p* < 0.05, ** *p* < 0.01, *** *p* < 0.001, **** *p* < 0.0001

**Supplementary Material 2**: Supplementary Fig. 2: Flow cytometric analysis of PD-L1 + cells in isolated neutrophils: representative plots of PD-L1 + neutrophils (A), and quantifications are depicted as percentage (B) and MFI (C)

**Supplementary Material 3**: Supplementary Fig. 3: The interaction network diagram of proteins and KEGG pathways. the blue dotted lines represent protein–protein associations; the white dotted lines represent the associations with KEGG pathways

**Supplementary Material 4**: Supplementary Fig. 4: Bacterial burden in the BALF, lung, liver, and spleen are shown in CFU (A). Representative histological images of lungs by H&E staining (B). Inflammation scores estimated from lung tissues with H&E staining (C). The counts of neutrophils in the BALF (D)

**Supplementary Material 5**: Supplementary Fig. 5: Flow cytometric analysis of PD-L1 + neutrophils in the lungs: representative plots of PD-L1 + neutrophils (left), and quantifications are depicted as percentage (upper right) and MFI (lower right)

**Supplementary Material 6**: Supplementary Fig. 6: Flow cytometric gating strategy of PD-L1 + neutrophils in the lungs (A) and PD-L1 + cells in isolated neutrophils (B)

**Supplementary Material 7:** Supplementary Table. 1: Neutrophil gene signatures

**Supplementary Material 8:** Supplementary Table. 2: qPCR primers

## Data Availability

Data will be made available on request.
