# Supplementary figures and images for: Fine particulate matter 2.5 induces susceptibility to Pseudomonas aeruginosa infection via expansion of PD-L1high neutrophils in mice
Source: Respir Res. 2024 Feb 14;25:90. doi: 10.1186/s12931-023-02640-x (PMC10865610; doi:10.1186/s12931-023-02640-x)

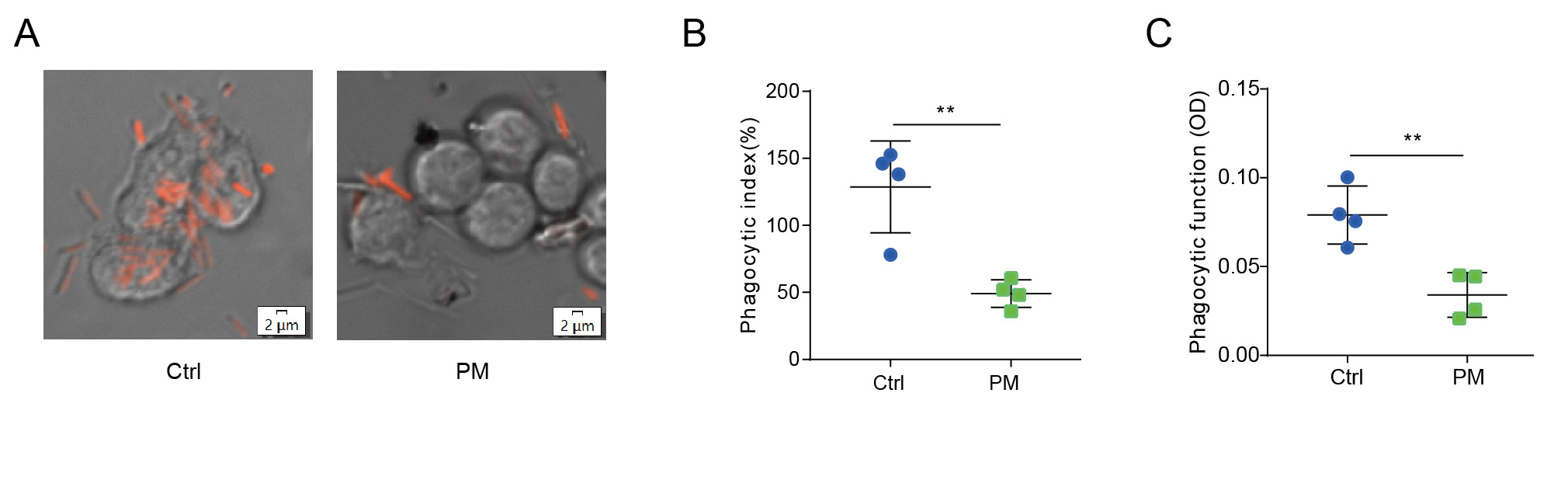

Supplement: Supplementary file 1 — Supplementary Material 1: Supplementary Fig. 1: Representative images of the phagocytosis process of mCherry-PAO1 by neutrophils. (A) Quantifications of (B) are depicted as phagocytic index. (C) Phagocytic function of neutrophils assessed by neutral red phagocytosis assay. data are shown as means ± SEM. * p < 0.05, ** p < 0.01, *** p < 0.001, **** p < 0.0001 [file 12931_2023_2640_MOESM1_ESM.jpg]

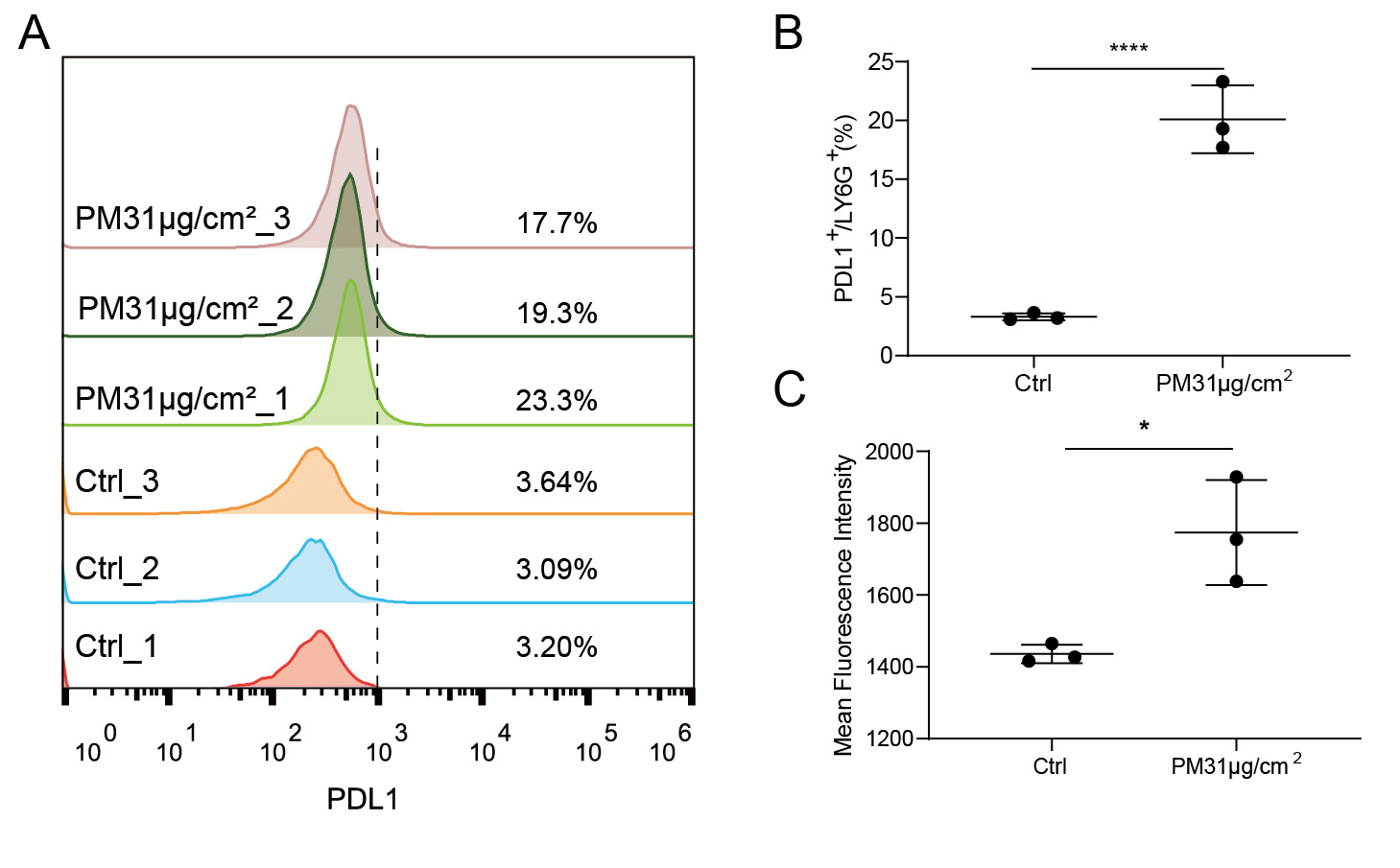

Supplement: Supplementary file 2 — Supplementary Material 2: Supplementary Fig. 2: Flow cytometric analysis of PD-L1 + cells in isolated neutrophils: representative plots of PD-L1 + neutrophils (A), and quantifications are depicted as percentage (B) and MFI (C) [file 12931_2023_2640_MOESM2_ESM.jpg]

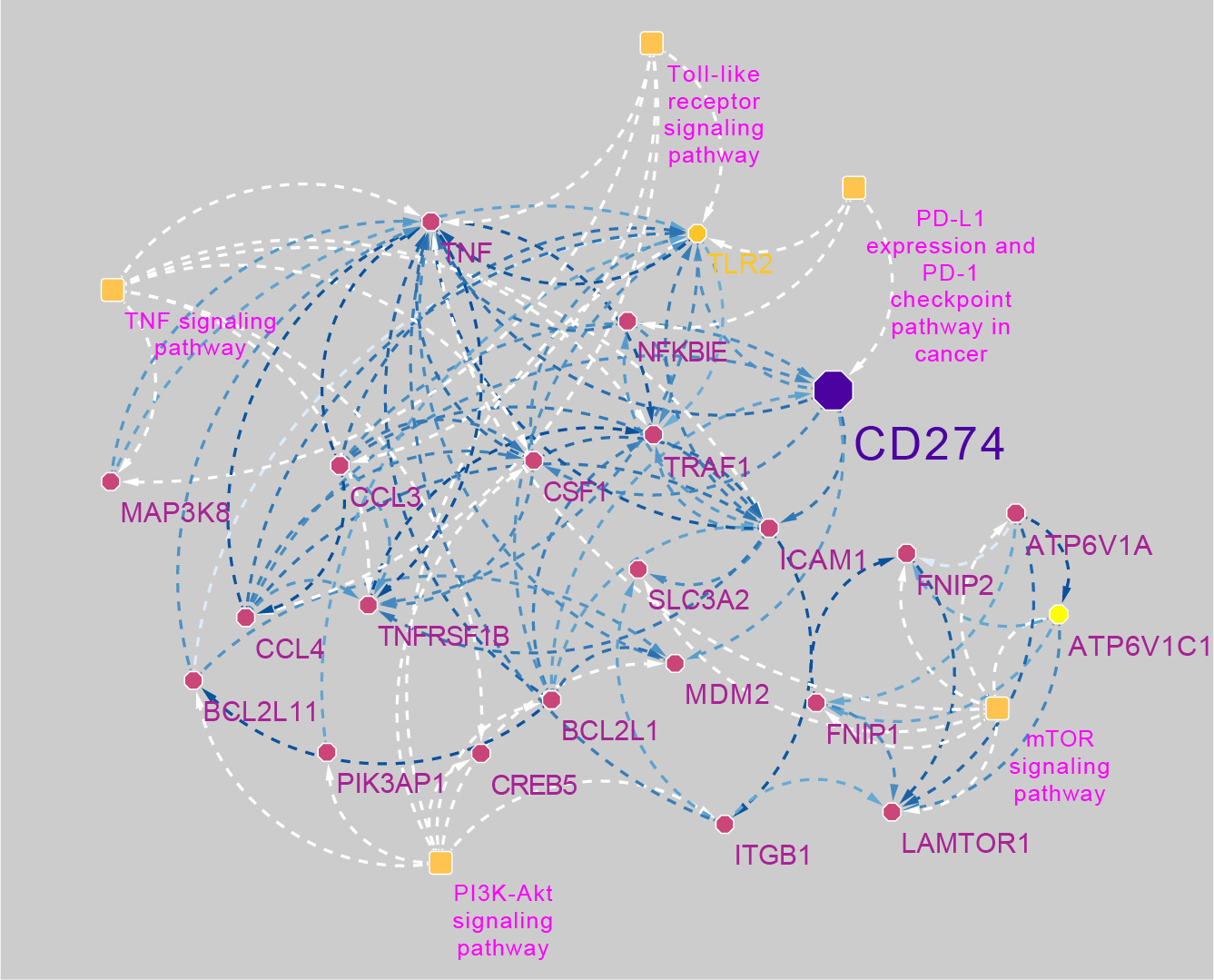

Supplement: Supplementary file 3 — Supplementary Material 3: Supplementary Fig. 3: The interaction network diagram of proteins and KEGG pathways. the blue dotted lines represent protein–protein associations; the white dotted lines represent the associations with KEGG pathways [file 12931_2023_2640_MOESM3_ESM.jpg]

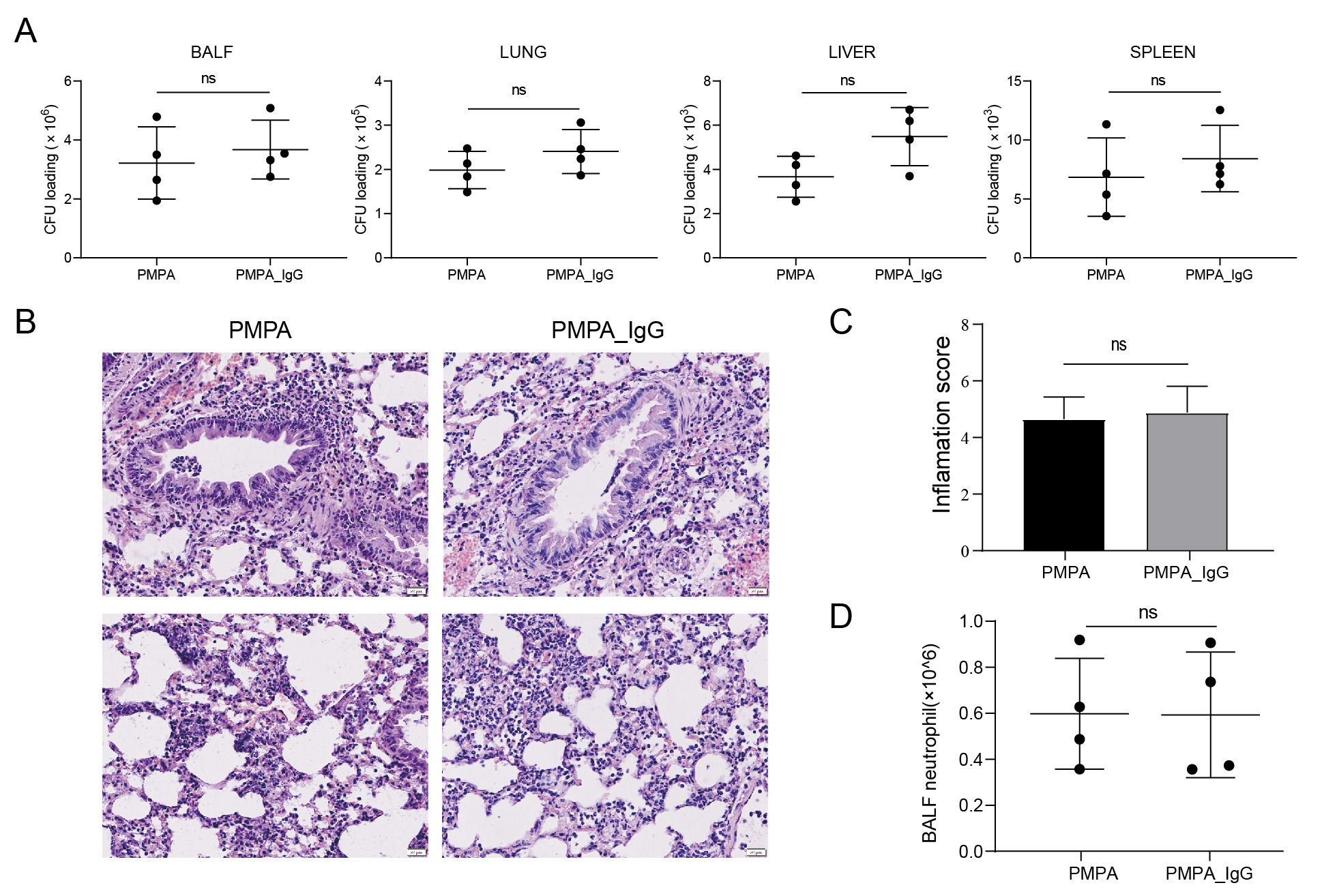

Supplement: Supplementary file 4 — Supplementary Material 4: Supplementary Fig. 4: Bacterial burden in the BALF, lung, liver, and spleen are shown in CFU (A). Representative histological images of lungs by H&E staining (B). Inflammation scores estimated from lung tissues with H&E staining (C). The counts of neutrophils in the BALF (D) [file 12931_2023_2640_MOESM4_ESM.jpg]

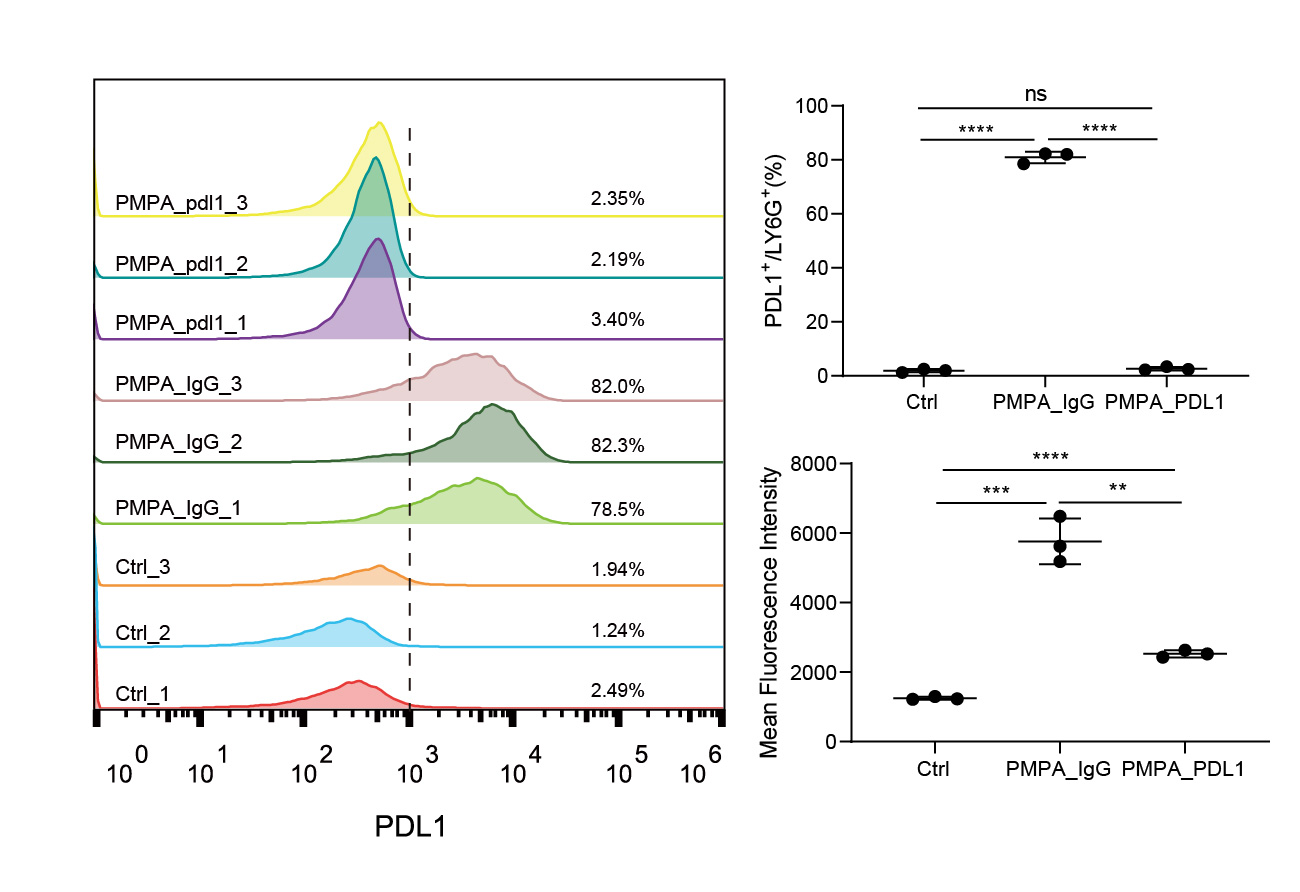

Supplement: Supplementary file 5 — Supplementary Material 5: Supplementary Fig. 5: Flow cytometric analysis of PD-L1 + neutrophils in the lungs: representative plots of PD-L1 + neutrophils (left), and quantifications are depicted as percentage (upper right) and MFI (lower right) [file 12931_2023_2640_MOESM5_ESM.jpg]

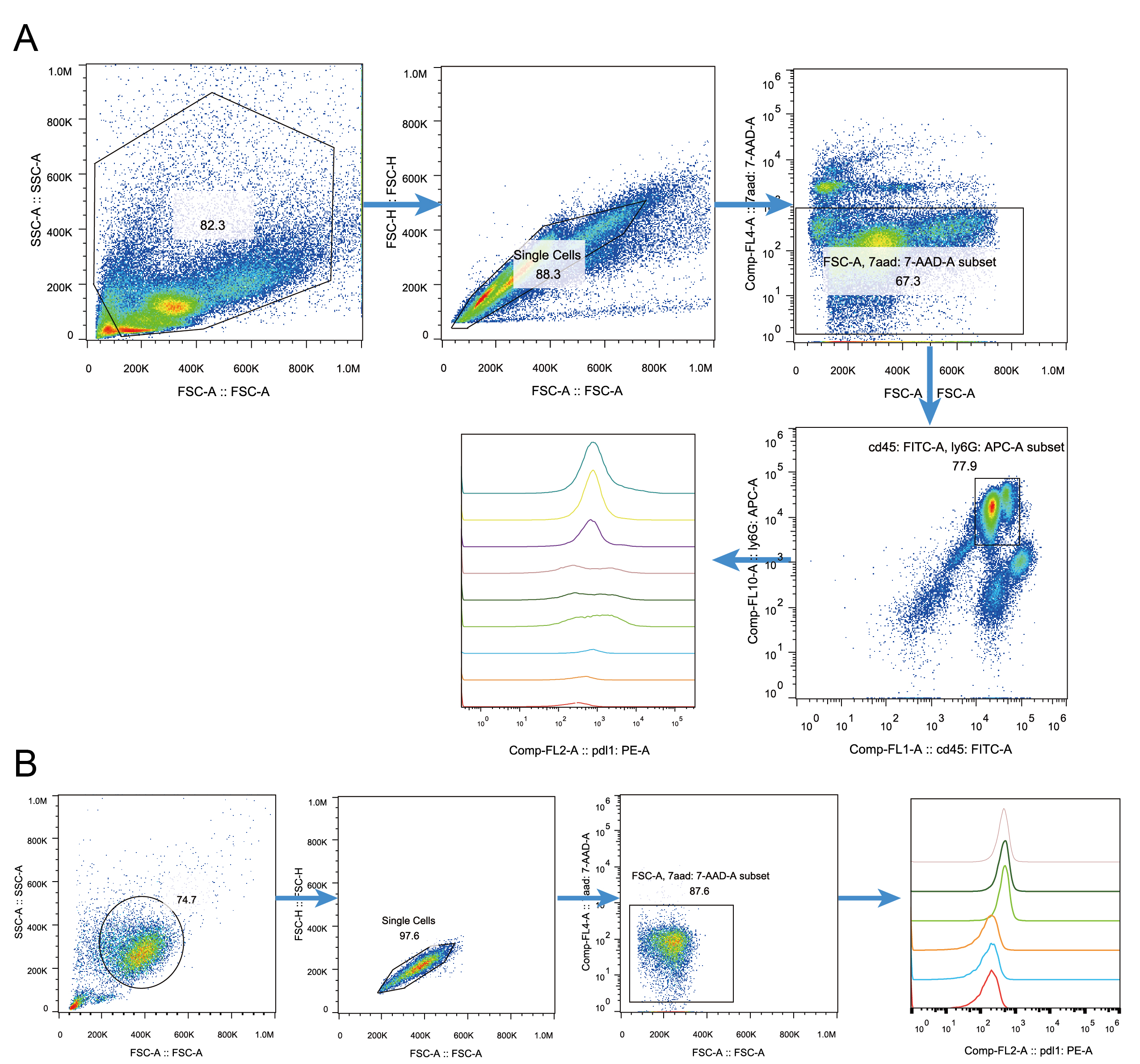

Supplement: Supplementary file 6 — Supplementary Material 6: Supplementary Fig. 6: Flow cytometric gating strategy of PD-L1 + neutrophils in the lungs (A) and PD-L1 + cells in isolated neutrophils (B) [file 12931_2023_2640_MOESM6_ESM.jpg]
